# “They don’t go by the law around here”: law enforcement interactions after the legalization of syringe services programs in North Carolina

**DOI:** 10.1186/s12954-022-00690-w

**Published:** 2022-09-27

**Authors:** Brandon Morrissey, Tamera Hughes, Bayla Ostrach, Loftin Wilson, Reid Getty, Tonya L. Combs, Jesse Bennett, Jennifer J. Carroll

**Affiliations:** 1 Department of Sociology and Anthropology, NC State University, 10 Current Drive, Campus, Box 8107, Raleigh, NC 27695-8107 USA; 2 Eshelman School of Pharmacy, University of North Carolina at Chapel Hill, Chapel Hill, NC USA; 3 Medical Anthropology and Family Medicine, Boston University School of Medicine, Boston, MA USA; 4 North Carolina Harm Reduction Coalition, Raleigh, NC USA; 5 Department of Medicine, Brown University, Providence, RI USA

**Keywords:** Police, Law enforcement, Drug policy, Syringe access, Harm reduction

## Abstract

**Background:**

In 2016, the US state of North Carolina (NC) legalized syringe services programs (SSPs), providing limited immunity from misdemeanor syringe possession when law enforcement is presented documentation that syringes were obtained from an SSP. This study explores the law enforcement interactions experienced by SSP participants since the enactment of this law.

**Methods:**

This study used a convergent, mixed-methods design consisting of structured surveys and semi-structured interviews with SSP participants in seven NC counties. Survey and interview data were collected simultaneously between January and November 2019. This survey was designed to capture demographics, characteristics of drug use, SSP services used, and past-year negative experiences with law enforcement (officer did not recognize SSP card, did not believe SSP card belonged to participant, confiscated SSP card, confiscated syringes, or arrested participant for possessing syringes). Semi-structured interviews explored lived experiences with and perspectives on the same topics covered in the survey.

**Results:**

A total of 414 SSP participants completed the survey (45% male, 54% female, 1% transgender or non-binary; 65% White, 22% Black, 5% American Indian/Alaskan Native, 8% some other racial identity). 212 participants (51.2%) reported at least one past-year negative experience with law enforcement. Chi-square testing suggests that Black respondents were more likely to report having experienced law enforcement doubt their SSP card belonged to them. Interview data indicate that law enforcement practices vary greatly across counties, and that negative and/or coercive interactions reduce expectations among SSP participants that they will be afforded the protections granted by NC law.

**Conclusion:**

Despite laws which protect SSP participants from charges, negative law enforcement responses to syringe possession are still widely reported. Evidence-based policy interventions to reduce fatal overdose are undermined by these experiences. Our findings suggest NC residents, and officers who enforce these laws, may benefit from clarification as to what is required of the documents which identify participants of registered SSPs where they may legally obtain syringes. Likewise, more thorough trainings on NC’s syringe law for law enforcement officers may be merited. Further research is needed to assess geographic differences in SSP participants’ law enforcement interactions across race and gender.

## Background

Despite wide recognition of the “opioid epidemic” as a pressing public health concern, opioid overdose fatalities in the USA continue to increase. Between December 2020 and December 2021, the USA saw an estimated 14.9% increase in overdose deaths [1]. Some states have implemented evidence-based interventions to combat increasing overdose deaths, including syringe service programs (SSPs). These programs can provide a variety of services, including distribution of safer substance use equipment; collection and safe disposal of used syringes; testing for infectious diseases; and linkage to care for those seeking treatment for substance use disorders, HIV, hepatitis C, and other health care concerns [2]. Through the provision of these services, SSPs can effectively prevent both overdose fatalities and new incidents of infectious disease [2].

In the US state of North Carolina (NC), the rate of overdose fatalities has risen nearly five-fold over the past 20 years, jumping from 6.5 deaths per 100,000 people in 2001 to 29.7 in 2020 [3]. In 2019, NC saw a 40% increase in overdose-related deaths, the largest single-year increase over the 22 years shown on NC’s data tracker [3]. In 2020, 93.2% of all overdose deaths in NC were opioid-involved [3]. As well, rates of polysubstance-involved deaths in NC have been steadily increaseing over time, accounting for over 60% of fatal overdoses in 2020 [3, 4]. That same year, Black and American Indian/Alaskan Native (AI/AN) persons experienced the largest increases (66% and 93%, respectively) in rates of fatal overdose in the state [3]. These deaths have prompted NC Division of Public Health to identify “turn[ing] the tide of North Carolina’s opioid and substance use crisis” as one of the state’s main public health goals [5].

**Fig. 1:**
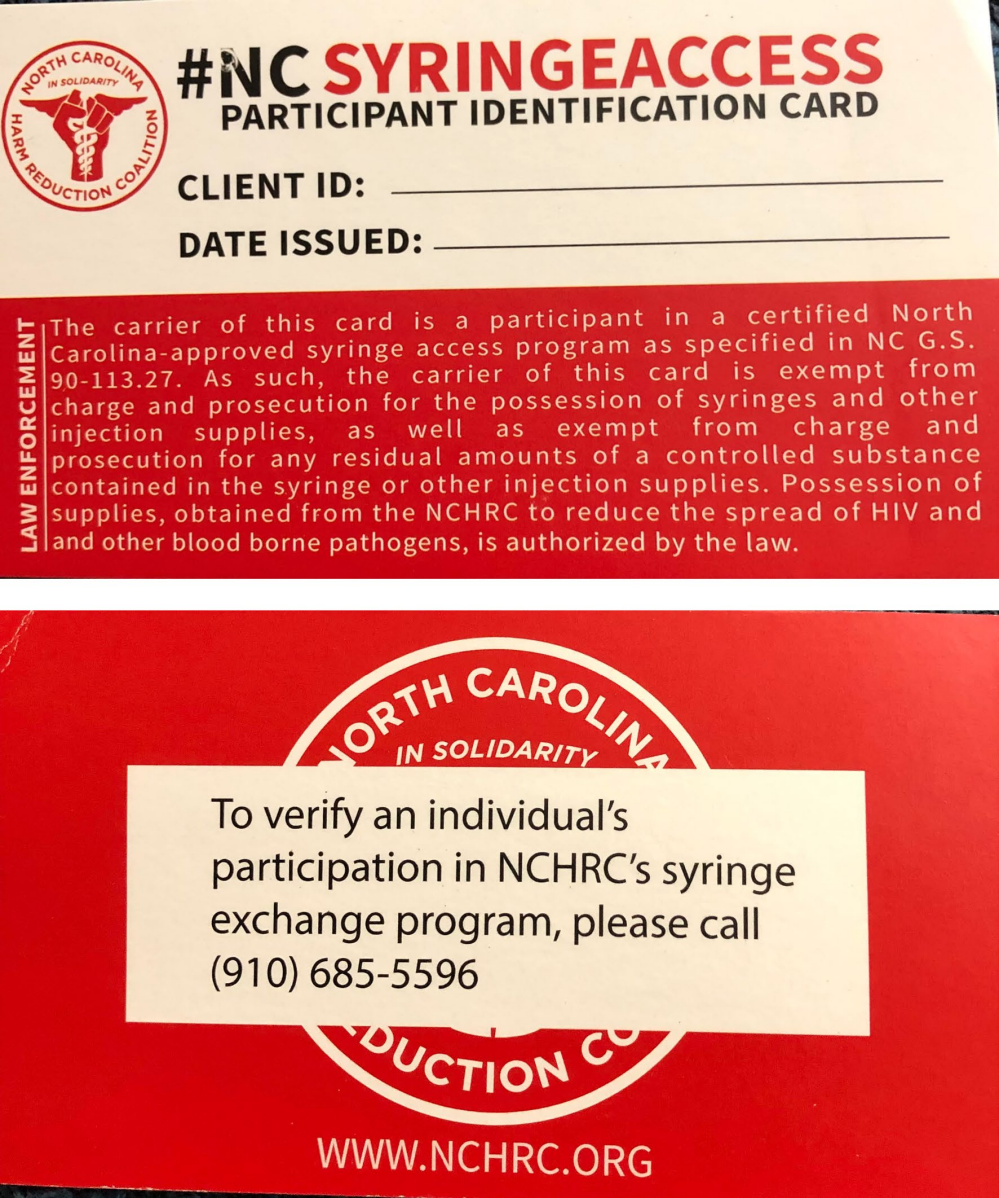
The SSP participant card used by North Carolina Harm Reduction Coalition at the time of research (front/back)

In July 2016, a syringe access law was enacted in NC (§ 90-113.27) to legalize the operation of “needle and hypodermic syringe exchange programs” (hereafter, SSPs) within the state [6]. This law provides limited immunity to participants, employees, and volunteers of NC SSPs from prosecution for possession of syringes (classified as drug paraphernalia under NC law) if they were obtained from an SSP operating pursuant to the law [6]. Notably, this immunity only applies “if the person claiming immunity provides written verification that a needle, syringe, or other injection supplies were obtained from a [legally-operating SSP]” [6]. According to NC’s Department of Health and Human Services, the syringe access law does “not specify format or required information for written verification,” and SSPs are free to create their own verification documents [7]. In practice, such documentation has, to the best of our knowledge, consistently taken the form of 3.5in by 2in participation cards that indicate an individual participates in a SSP registered with the state. Some programs mark these cards with a unique identifier for each participant; other programs do not (see Fig. 1).

Law enforcement actions shape health outcomes among people who use drugs (PWUD). Research has demonstrated that fear of arrest can drive unsafe injection practices (increasing the risk of HIV, hepatitis, and bacterial infection [8]) and deter community members from calling first responders (primarily emergency medical services, but police are also regularly dispatched to emergency calls) during an overdose emergency, with potentially deadly consequences [9, 10]. Similarly, participation in SSPs may be deterred by proactive police practices, such as heightened foot patrols, increased police stops, and increases in arrests around the areas where SSPs operate [11–13]. Similar negative law enforcement interactions are common among those who utilize SSP services. In a 2008–2009 nationwide survey of SSP managers conducted, 43% reported that clients faced harassment by law enforcement at least monthly, and 31% reported that clients had injection equipment confiscated by law enforcement at least monthly [14].

Race and gender are categories of social identity and social distinction that further influence SSP participation and law enforcement interactions in the context of substance use and SSP utilization. Historically, White persons who inject drugs have had higher odds of accessing sterile syringes from SSPs compared to their Black counterparts [15]. Racial inequity varies across service modalities as well. Some research suggests that Black individuals are more likely to use mobile SSP services compared to a fixed location SSP [16]. Research suggests that women are more affected by stigma concerning drug use, as well as concerns about state welfare agencies taking action to separate children from their parents, leading to less willingness to participate in SSPs [17]. These concerns may be compounded among women from racialized minority groups, as Black and AI/AN children are disproportionately over-represented in the foster care system, and as family reunification after the forced removal of a child is significantly less common among AI/AN families [18, 19].

Racialized inequities in access to harm reduction and health care services are best understood in the context of the long history of oppression wrought by agents and institutions of law enforcement on Black communities in the USA. Slave patrols, common across the American South throughout the 1800s, were early police systems designed to control the Black population [20, 21]. After the American Civil War ended in 1865, law enforcement groups continued to target Black persons through enforcement of racially-discriminatory laws with the intent of reestablishing slavery under a convict-leasing system [22, 23]. Throughout the 1900s, law enforcement worked alongside anti-Black racists from White communities to maintain segregation in both public spaces and residential areas [24]. Such racial segregation created predominately Black and/or Hispanic neighborhoods which were then (and continue to be) under-resourced and overpoliced [25, 26]. When President Nixon declared a War on Drugs in 1971, it was done with the express purpose of criminalizing Black Americans and fracturing Black communities through targeted police actions implemented under the banner of public safety [27, 28]. Contemporary policing in the USA continues to disproportionately disadvantage Black communities due in large part to the laws, institutions, and policing cultures generated—and often still justified–by the War on Drugs and the framing of substance use by law enforcement leaders as a fundamentally criminal problem [22, 29, 30]. Today, Black individuals are overrepresented at virtually every level of criminal-legal contact [31], including arrests for drug offenses [32].

Unsurprisingly, prior research confirms that racialized identity differentially affects the experiences of PWUD when interacting with law enforcement. Increased police activity around SSPs is associated with reduced SSP participation among Black and male participants (compared to White and female counterparts) and increased needle-sharing [8, 33]. A national survey of SSPs found that programs predominantly serving Black or Hispanic participants were more likely to report arrests and harassment of their participants [14]. In an effort to ensure full implementation of SSPs and other harm reduction services, researchers have called for better data collection as it related to law enforcement experiences among PWUD [34]. A previous study of SSP participants in western NC found that individuals who participated in SSPs reported, on average, nearly double the frequency of being stopped and searched when compared to those who got syringes elsewhere [35]. However, to the best of our knowledge, SSP participants’ interactions with law enforcement officers across NC as they pertain to officers’ implementation of the state’s syringe access law since its enactment in 2016 have not been systematically assessed.

The purpose of this mixed-methods study is to explore SSP participants’ interactions with law enforcement officers—especially as officers take specific actions within the framework of the 2016 syringe access law—and assess the association between negative police interactions and the race and gender of SSP participants after SSP legalization in NC.

## Methods

### Study design and setting

We conducted a cross-sectional, mixed-methods study consisting of a structured survey and semi-structured interviews with individuals accessing services at seven unique SSP sites operated by the NC Harm Reduction Coalition (NCHRC) in Cumberland, Durham, Haywood, Johnston, New Hanover, Vance, and Wake counties of NC, respectively.

### Study population and recruitment process

Eligible participants included persons who were at least 18 years old at the time of recruitment, currently receiving harm reduction services from one of the seven SSPs included in the study, and able and willing to provide informed consent. Recruitment took place between January and November 2019 with the assistance of trained NCHRC staff. Specifically, NCHRC staff who provide SSP services within at least one of the seven counties informed SSP participants of the study during the course of normal SSP operations. If participants indicated interest, NCHRC staff directed them to speak with researchers, who were positioned in a location nearby yet away from the area of service provision (somewhere mutually agreed upon with local SSP staff) that offered a reasonable expectation of privacy and minimized the disruption of services. If participants approached the researcher and expressed interested in participating, the informed consent process and data collection immediately followed. Consent was obtained verbally. To reduce perceptions of coercion, program participants were only told about the study after they had received services and concluded their formal interactions with NCHRC staff.

### Data collection: surveys and interviews

All participants who met eligibility conditions and verbally provided informed consent were asked to complete a self-administered, written survey. Upon such request by any participant, the researcher would administer the survey verbally and record participant answers in writing. Participants were offered $20 gift cards as incentive for completing the survey. Some who completed the survey were also invited to participate in a semi-structured interview. Interview participants were selected with a purposive or range-maximizing sampling strategy [36] attempting to maximize variation in participant age, race/ethnicity, and gender in order to capture insight that might explain trends in the survey data and identify (for purposes of future research) any domains deemed important by participants that the survey was not designed to capture. Participants received an additional $40 gift card as incentive for participating in an interview. All interviews were semi-structured and included the following a priori domains: substance use behaviors (substances used, frequency of use, route of administration); treatment history; history of overdose; perceptions of SSP services; additional healthcare, treatment, or service needs; and previous interactions with law enforcement and the criminal justice system.

### Survey measures

The survey asked participants for demographic information; information about their history with substance use and treatment for substance use disorder; and about past year interactions with law enforcement. Demographics data collected included a single question for self-reported race and ethnicity (as listed on the survey: Asian, Black, Caucasian, Hispanic, Native American, Multiracial, and Other; participants were asked to check all that apply); self-reported gender (as listed on the survey: male, female, transgender, intersex, non-binary, other; participants were asked to check all that apply), and age range (18–25 years; 26–30 years; 31–35 years; 36–45 years; 46–55 years; 56–65 years; and 66 years or more).

To explore SSP participants’ interactions with law enforcement, survey takers were also asked to indicate whether or not they had experienced each of the following events in the past year: (1) “Showed exchange card to law enforcement but they [law enforcement] weren’t familiar with the syringe exchange [SSP] law”; (2) “Law enforcement didn’t believe the exchange [SSP] card belonged to you”; (3) “Law enforcement confiscated your exchange [SSP] card”; (4) “Law enforcement confiscated supplies you got from the exchange [SSP]”; and (5) “Arrested for supplies you got from the exchange [SSP].” Answers were recorded as dichotomous (yes/no). Respondents could also check a box indicating they experienced some “Other” type of law enforcement interaction and then provide additional details as an open text response.

Two composite variables were produced for this analysis. The first composite variable captures an affirmative answer to any of the five questions (“Other” excluded). The second composite variable captures an affirmative answer to questions of coercive police interactions: specifically, confiscation of SSP card, confiscation of supplies, arrest for SSP supplies. Both composite variables were coded as dichotomous.

### Analysis

*Survey data* Descriptive statistics were generated for demographic variables, substance use characteristics, and reported experiences with law enforcement. Chi-squared tests were used to assess the difference in likelihood of reported negative experiences with law enforcement across categories of race (White and Black), gender (female), and of geographic location (county, when indicated by descriptive trends as described in Sect. 3.2). We report on trends among White and Black participants but not participants who claimed other racial or ethnic identities, because the participant populations representing these other identities were too small for analysis. All analysis was conducted on R V.4.1.2 (2021, RStudio Team, Delaware Public Benefit Corporation, Boston, MA, USA).

*Interview data* All interviews were audio recorded and transcribed for analysis. Interviews were, first, deductively coded by hand by one coder [JC] to identify all excerpts relating to law enforcement and law enforcement interactions. These excerpts were subsequently re-analyzed by hand and subject to inductive, open-coding exercises by two coders [BM and JC] to better characterize the disposition of reported law enforcement interactions (i.e., resulted in arrest, resulted in confiscation of supplies, etc.) and tone or lived experience of law enforcement interactions (i.e., officers were reportedly lenient, officers were reportedly aggressive in searching, etc.). After independently coding, coders met to discuss results and reconcile any differences. We then re-examined these inductive codes in light of findings from our analysis of survey data and noting whether interview data appeared to explain those findings, appeared to contradict those findings, or evidenced elements of law enforcement interactions not captured in the survey. The findings presented here emerged in this final stage of analysis.

## Results

### The study sample

A total of 414 individuals participated in the survey (see Table 1). Survey respondents were majority female (53.6%) and White (63.2%). The second largest racialized identity reported was Black (21.5%). Each of the other identified racial categories constituted, separately, less than 5% of the total sample. Only 5 participants (1.3%) reported a gender identity other than exclusively male or exclusively female. Approximately 85.0% of participants (*n* = 352) reported regular (at least monthly) opioid use and 9.4% (*n* = 39) reported frequent (“everyday” or “a few times per week”) opioid use. Regular stimulant use was reported by 78.7% of participants (*n* = 326) and regular benzodiazepine use by 42.0% (*n* = 174). Among all participants, nearly three-quarters (73.7%, *n* = 305) reported using one or more substances by injection.

**Table 1:** Participant demographics

	*n* (%)
Reported experiences	
Any Negative interaction	212 (51.2)
Any Coercive interaction	113 (27.3)
County	
Durham	80 (18.6)
Wake	39 (9.1)
New Hanover	49 (11.4)
Cumberland	61 (14.2)
Vance	103 (24.0)
Johnston	17 (4.0)
Haywood	65 (15.1)
Age	
18–25	40 (10.3)
26–30	84 (21.7)
31–35	65 (17.6)
36–45	97 (25.1)
46–55	60 (15.5)
56–65	37 (9.6)
66+	4 (1.0)
Race	
White	262 (64.9)
Black	89 (22.0)
Native American	19 (4.7)
Hispanic	9 (2.2)
Asian	4 (1.0)
Multiracial	8 (2.0)
Other	13 (3.2)
Gender	
Male	177 (45.2)
Female	210 (53.6)
Transgender, Intersex, or Other	5 (1.3)

Twenty survey participants also participated in semi-structured interviews. Interview participants, by self-report, were 65% female, 80% White, and 20% Black or mixed-race. At least three interview participants were recruited from each of the following counties: Cumberland, Durham, Haywood, New Hanover, Vance, and Wake. Data collection in Johnson County ended before interview recruitment began due to unforeseen staffing changes.

### Quantitative findings

**Fig. 2:**
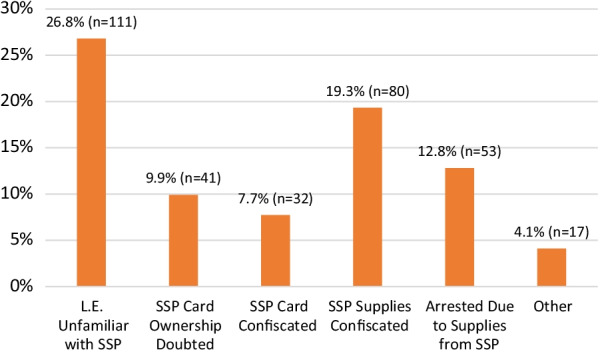
Negative law enforcement experiences reported by SSP participants

Approximately half (51.2%, *n* = 212) of all survey respondents reported having experienced at least one of the five negative law enforcement interactions indicated on the survey in the past year (see Fig. 2). The most common experience, reported by 26.8% of respondents, was a law enforcement officer stating they were unfamiliar with SSP identification cards or the syringe access law that provided immunity from paraphernalia charges when the card was presented. Nearly 1 in 5 respondents (19.3%) reported that an officer confiscated syringes obtained from the SSP, and 12.8% reported that an officer placed them under arrest for possession of syringes obtained from an SSP. A total of 17 respondents (4.1%) marked “Other” when asked about law enforcement interactions. Open text responses elaborating on the nature of these “other” experiences included descriptions of general harassment (for example, “law enforcement threatened to charge me for supplies I had gotten from the exchange”), being ticketed for other offenses after showing the SSP card (such as driving in excess of the posted speed limit), confiscation of the overdose-reversal medication naloxone (also distributed by NC SSPs), and post-release violations.

More than half (55.1%) of Black respondents reported experiencing at least one of the negative law enforcement interactions indicated about in the survey in the past year. This rate is higher than that of all other participants (50.1%); however, chi-square analysis showed this difference was not significant. Additionally, the proportion of participants identifying as male and participants identifying as female reporting at least one negative law enforcement experience in the past year (52.5% and 49%, respectively) was also not significantly different.

**Fig. 3:**
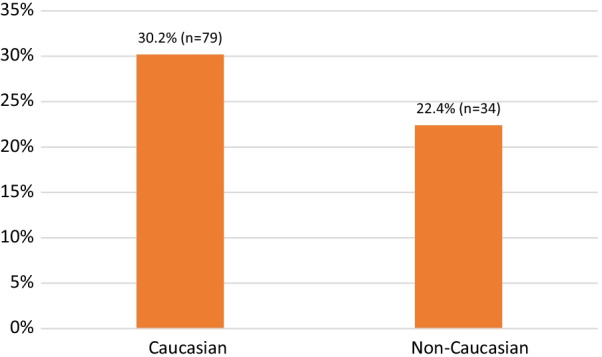
Reports of Coercive Law Enforcement Interactions by Race*. *Includes reports of one or more of the following experiences in the previous year: confiscation of SSP card, confiscation of supplies, and arrest for SSP supplies

**Fig. 4:**
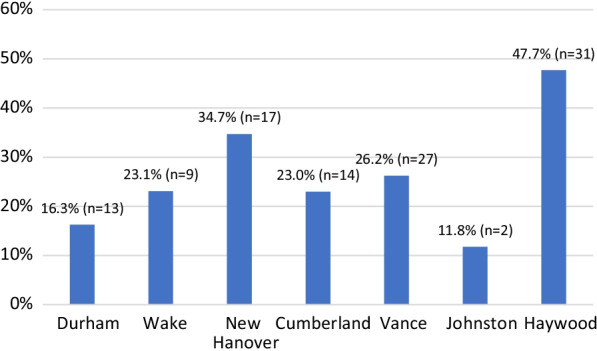
Reported incidents of coercive law enforcement interactions*. *Includes reports of one or more of the following experiences in the previous year: confiscation of SSP card, confiscation of supplies, and arrest for SSP supplies

Chi-squared analysis found that White participants, compared to respondents of all other races, were significantly (*p* = 0.015) more likely to report a coercive law enforcement interaction (i.e., confiscation of SSP card, confiscation of syringes obtained from an SSP, or arrest for possession of syringes obtained from an SSP; see Fig. 3). Reports of coercive experiences were markedly more common in Haywood County (47.7% of respondents) compared to all other counties (range 11.8–34.7%; see Fig. 4). In Haywood County, 81.5% of respondents are Caucasian. This raises the clear possibility that this finding is a result of unique policing practices in Haywood County—a hypothesis that is further supported by qualitative findings (discussed in Sect. 3.3.2).

**Fig. 5:**
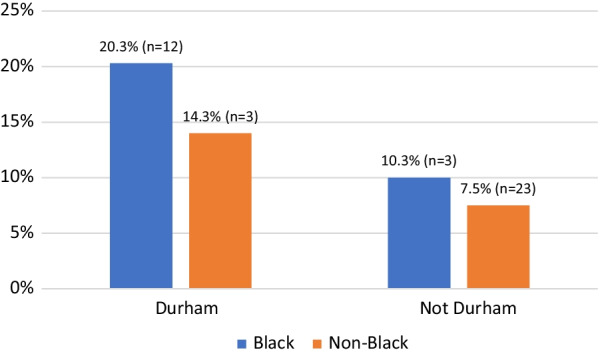
Reports of law enforcement doubting SSP card ownership, by race and county

Black respondents were significantly (*p* = 0.019) more likely to report experiences in which law enforcement officers did not believe their SSP card belonged to them compared to respondents of all other races. Of note, 68.6% of all Black respondents in this study were recruited in Durham County, raising the possibility that this difference is a result of law enforcement practices in Durham. However, as shown in Fig. 5, a larger proportion of Black participants, compared to participants of all other races, reported officers doubting ownership of their SSP card in regions beyond Durham County (10.3 vs. 7.5%, respectively) as well as within Durham County (20.3 vs. 14.3%, respectively). This suggests, but does not conclusively indicate, that this trend may not be unique to Durham County.

**Fig. 6:**
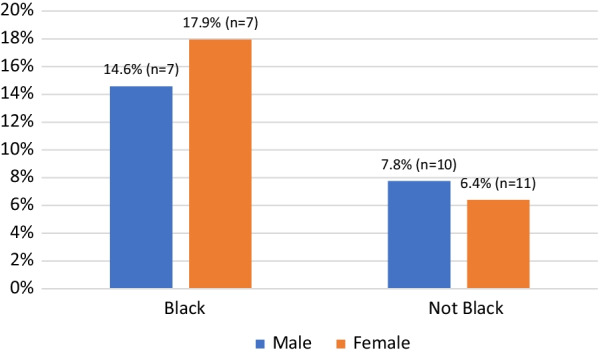
Reports that law enforcement doubted SSP card ownership, by race and gender

Nearly one-fifth (17.9%) of Black women who participated in the survey reported experiencing an incident in which a law enforcement officer did not believe their SSP card belonged to them; further, Black women were more likely to report this specific experience compared to all other participants. This trend approached, but did not fully reach, statistical significance (*p* = 0.083). Of note, as illustrated in Fig. 6, the relationship between race, gender, and reporting that an officer did not believe a participant’s SSP card belonged to them is reversed for non-Black respondents. In other words, women in our sample who reported any racial identity other than Black were less likely to report law enforcement doubting the ownership of their SSP card compared to non-Black, male persons. This trend was visible in our sample, but not significant.

Due to sample size, we are unable to statistically investigate these trends across race, gender, and county further.

### Qualitative findings

In interviews with SSP participants, four major themes related to law enforcement interactions emerged: (1) variability in use of discretion by law enforcement; (2) the negative tone of police encounters; (3) doubt among some participants that SSP cards would afford them meaningful protection from criminal or other practical consequences during law enforcement interactions; and (4) coercive police practices. We discuss these themes in turn below.

#### Variation in use of discretion by law enforcement

In interviews, some respondents reported experiences in which law enforcement officers were familiar with and behaved in conformity with the provisions of NC’s 2016 syringe access law. For example, a Black man from Durham County described an incident during which he and two associates were briefly detained after police officers found syringes in their car:

[I said to the officer] “Take this card, read it, they belong to me.” “Not all of these—all of these don’t belong to you?” It was the corporal, a Durham Police Department corporal. He tried to be a hard ass, but then he read the card and he was like, “Uh, you taking responsibility for all these needles that’s in this car?” I was like, “Officer, they belong to me. Leave [the other woman in the car] alone.” And they didn’t charge her with that.

Notably, this interviewee described this interaction as typical of officers in the Durham County area, which is consistent with other interviews with persons from Durham County.

Other interviewees reported different variations in the use of discretion during law enforcement interactions, with some officers reportedly confiscating syringes and others stating a variety of reasons for allowing SSP participants to keep them. In Vance County, several interviewees reported that law enforcement do not take syringes found during traffic stops. One woman said that officers had stated, *“[I] don’t want that shit*.” In contrast, a woman in New Hanover reported that, when stopped by law enforcement, officers will typically *“ask you if you have anything on you and then, of course, I [tell] them what I [have] on me and they make me, you know, give them the syringe*.”

A woman in Cumberland County reported experiencing a variety of inconsistent discretionary actions by law enforcement during these interactions. She recalled:

We got pulled over, it was, like, bad tags, you know? [The officers] wanted to search the vehicle. And I was like, “Okay, well, I’m gonna let you know right now that I have them [syringes].” And they asked where they are, and then they – they don’t take them, sometimes they take them. Sometimes they don’t take them, but sometimes they do take them and they throw them away. And other times they just put ‘em back and they leave ‘em.

As this participant notes, variation in law enforcement responses to the possession of SSP supplies is reported to occur not simply across counties or law enforcement jurisdictions, but *within* jurisdictions—and within the lived experiences of individual SSP participants as well. Consequently, some participants faced not only coercive law enforcement action, but coercive action that was sporadic and unpredictable.

#### Drug use and the tone of law enforcement interactions

Some interviewees reported that interactions with law enforcement officers tended to be overwhelmingly negative in tone or attitude. For example, a younger White woman from Vance County described officers regularly seeking cause for arrest due to drug residue in syringes or for possession of harm reduction supplies obtained from the SSP that are not explicitly covered under the NC syringe access law:

[Police will] test [your syringe], to see if there’s heroin residue in it. And they’ll try to charge you with it, if the heroin—not the needle, because they can’t charge you with the needle. But if there’s residue…They can’t charge you with a needle, but if we get pulled over because we sniff it with a cut straw, they’re going to charge us with a cut straw. I’ve [been] charged with a straw before…

Such descriptions were common across interviews. 

A young White woman from Haywood County also described being regularly subject to aggressive or intrusive searches. She described one kind of typical interaction as follows:

There’s been plenty of times when we’ve been pulled over for no reason whatsoever. And they make the reason up or saying that you’re going five miles over the speed limit, which you know you weren’t because you seen a police officer right behind you, and you weren’t going over the speed limit. Or you went over the line, things like that. Then they ask if there’s anything in the car. You say no. They ask if you can search, and you say no. And they just bring a dog out and say the dog hits on the car and they end up searching the car.

This interviewee reported that officers consistently claimed to have found cause for pretextual vehicle searches—the validity of which she openly contested—rendering moot her right to refuse consent. The same woman later noted:

I think’ cause we’re drug users so they just – they’re automatically judgmental towards us right off the bat, you know, so no matter what we say or anything, it’s a very judgmental attitude towards it…

In other﻿﻿ words, from her perspective, officers respond more negatively to any information provided by her, simply due to her status as a PWUD. Though some reported officers declining to interfere with participants in possession of syringes obtained from an SSP, many interviewees described interactions similarly characterized by antagonism and criminalization, wherein law enforcement officers sought to locate a chargeable offense not covered by the state’s syringe access law.

As illustrated by this and other incidents (such as those described in Sect. 3.3.1, above), reports of especially antagonistic interactions with law enforcement were relatively common in interviews conducted in Haywood County. That pattern contrasts sharply with descriptions of law enforcement interactions in, for example, Durham County, where the SSP serves a large population of Black residents. A middle-aged Black woman from Durham reported that “*[The] cops have never stopped me,*” and that local law enforcement “*don’t bother [SSP participants].*” A second Black woman from Durham described law enforcement as “*…pretty good around here…I mean they patrol [the neighborhood] pretty hard sometimes, but for the most part, I mean, they just do their jobs.*” Though not conclusive, these differences across counties provide support for our hypothesis that policing practices unique to Haywood County (a majority White county) are producing our finding (described in Sect. 3.2, above) that White SSP participants are more likely to have SSP supplies confiscated or be placed under arrest for possessing those supplies.

#### Mixed views on the utility of SSP identification cards

Some interview participants were fully enrolled participants of their local SSP and carried SSP identification cards; a few, however, were not. Some participants—including some who did and some who did not carry these cards—expressed skepticism about the practical protections offered by carrying SSP participant cards. Across counties, interviewees reported law enforcement rejecting the SSP card as a form of legal protection for people with whom they interact. In Haywood, a White man in his early 20s reported recently being arrested and having his supplies confiscated: “*I saw [the police officer] find some syringes… And he looked at me with this [expression] and was like ‘Ain’t nobody gives a shit about that card’ and flicks it out of my fingers.”* This was an uncommon but particularly egregious example from our data. The interviewee made it clear that the officer appeared to be familiar with the syringe access law and appeared to understand the protections the card was supposed to afford, yet knowingly disregarded those state policies and chose to place this young man under arrest anyway.

Several respondents in other counties also stated that the SSP identification cards do not provide adequate protection from further criminal-legal system involvement. In Wake County, a White woman in her late 30s summarized this perspective when she stated the following:

There’s no point in you carrying the card if you’re still going to get in trouble…Nine times out of ten, if you get charged and you go in front of a judge, that’s [the card’s] not gonna hold no weight and it’s [the charge is] gonna stick, you know? You’re still gonna get in trouble.

This interviewee questioned the benefits of carrying the SSP card if the protections it should legally afford were not recognized or upheld by either law enforcement or the courts. Notably, this interviewee felt that one pressing concern with the new syringe access law was that law enforcement officers were inadequately educated about its provisions, noting, “*they need to make to where it's [changes to the syringe access law] known to the whole and to everybody in the law enforcement*.”

Though it is possible to have possession charges dropped once the SSP participant appears before a judge, interviewees reported suffering several additional consequences while undergoing that process. A woman from New Hanover described this issue as follows:

I don’t think the law takes the cards and stuff as seriously…Not me personally, but people I’ve been with... basically [would] get arrested anyway. And of course it gets dropped when it goes to court, but it’s the process of getting there. And if you don’t have anybody to bond you out, you know, sometimes North Carolina takes their time…It [could] be anywhere between a week to 30 days depending on if they’re pushing off your court date or whatever. And sometimes they do it just to fuck with you, simply. You know? They know it’s gonna get dropped.

In other words, this interviewee argues that the process of undergoing arrest only to have charges eventually dropped is perceived as a punitive action by the law enforcement officers who initiate it. As noted, even this corrective process can be particularly disruptive to participants, resulting in jail stays that keep them away from family, work, and other obligations for weeks at a time.

#### Coercive police practices

Some respondents described law enforcement attempting to coerce SSP participants to cooperate with police investigations. Several reported officers saying they could make drug charges *“go away,”* if respondents would cooperate with law enforcement on other matters. As a White woman from Vance County described:

*"*You wanna go to work for me?" That’s what they ask. And the same exact detective—this was two different instances…[A]nyways, the cop called us, and had me and [my friend] up [at the station] and told us we were facing like four different felony charges…all of which was bullshit, all bullshit, straight bullshit, and [said] he could make those go away if we do some work for him.

She later shared her belief that these were empty threats fabricated for coercive purposes, saying, “*We walked out and didn’t tell anybody, and we never got served a warrant.*”

Similarly, a White woman in her late 30s from Cumberland County described a situation in which she and her boyfriend were visited by an officer at home after a traffic stop when law enforcement found syringes in their vehicle:

They had like the drug enforcement people come out to talk to me and my boyfriend. And, you know, they wanted to work some kind of deal out where I would turn people in or whatever. And I’m like, “You’re gonna get me shot out here, because…everybody can see you talking to me, and this does not look good.”

This interviewee framed the officers’ request as an offer for a mutually beneficial exchange in which she would get leniency for paraphernalia charges (from which she technically already had immunity as an SSP participant) in exchange for providing law enforcement with information about others involved in the drug trade. Yet, she voiced her suspicion that the practice of pressuring SSP participants was related to the fact that law enforcement “*don’t want [the SSP] here.*”

A White woman in her 20 s from New Hanover County described other coercive language used by officers who responded to an overdose. This respondent had been staying in a hotel with friends when the overdose occurred. She called 9–1-1, and responding officers noted the presence of illicit substances on the scene. She recalled:

I was like, doesn’t the Good Samaritan Law mean that as long as it’s under a certain [amount] you guys can’t prosecute me for this? And they were kind of like, "well, yea as long as you don’t give us any trouble…”

While this individual is not talking about protections under the syringe access law specifically, this instance demonstrates how criminal-legal protections extended to PWUD—in this case, by NC’s 9-1-1 Good Samaritan Law—may be framed by law enforcement. Again, leveraging the threat of arrest or charges, even if that punishment is out of accordance with the syringe access law as it is written, allowed the officer to attempt to coerce cooperation.

## Discussion

This mixed-methods study explored interactions between SSP participants and law enforcement in the wake of changes to NC law to legalize SSPs and provide limited immunity from prosecution for the possession of syringes obtained from an SSP operating pursuant to state law. Our survey data show that negative interactions with law enforcement officers pertaining to the implementation of this syringe access law are common among SSP participants despite the ostensible intention of the law to mitigate precisely these tensions in precisely these officer-citizen exchanges. Additionally, we found that Black SSP participants were significantly more likely to report law enforcement officers doubting the ownership of their SSP participant card compared to all other participants. In our sample, Black women were the most likely to report law enforcement officers doubting ownership of their SSP card compared to all other groups; this difference approached but did not achieve statistical significance, possibly due to small sample size.

Interview data indicate that the implementation of NC’s syringe access law varies greatly in practice across—and sometimes within—different regions of the state, with some officers showing little interest in taking syringes away from an SSP participant after documentation is produced and others preferring to confiscate and dispose of SSP syringes. Some of these practices, such as confiscation of SSP supplies, are not expressly prohibited by NC’s syringe access law, though they arguably contradict the spirit and undermine the public health benefits of that law. Regular, negative experiences with law enforcement have contributed to doubts among some SSP participants that the law or the SSP participant cards offer them meaningful protection from arrest and incarceration, regardless of whether that incarceration results in criminal prosecution for possession of paraphernalia. Considered together, these results provide further support that—even in a sociolegal environment which provides protections for SSP participants—law enforcement practices remain a serious impediment to syringe access and, therefore, the health and well-being of PWUD.

These findings align with prior work demonstrating that “disconnects” between policy change and lived experiences of SSP participants stems from the “street-level implementation” of said policy change [34]. One explanation for this common challenge to policy implementation is the role of law enforcement officers as, in the words of political scientist Michael Lipsky, “street-level bureaucrats” [37]. A street-level bureaucrat framework recognizes that law enforcement officers have “extensive autonomy in policy implementation,” such that they can choose “which policy to apply and how” [38]. Changes to the law are important, but, as stated by one interviewee in this study, “*they don’t go by the law around here*.” To combat this, state policymakers could develop plans to ensure a more robust implementation of policy changes at the local level. While some law enforcement agencies have partnered with community organizations to improve interactions among SSP participants and law enforcement officers, policymakers should not fully rely on overworked and underfunded community organizations to accomplish these administrative tasks.

Though our sampling strategy precludes any conclusions about frequency or geographic trends in these differences in use of discretion by law enforcement, the wide variability in reports collected suggests that the decision of whether to confiscate syringes may be, in some cases, guided by department policies or norms and, in other cases, may truly be a decision made at the discretion of individual law enforcement officers. NCHRC has undertaken efforts to provide meaningful education about the syringe access law to law enforcement agencies across the state. Nevertheless, NCHRC could not ensure contact with every sworn officer, and, aside from those efforts, NC law enforcement received little official communication about the law and how it would affect the day-to-day activities of law enforcement officers. According to NCHRC staff, some law enforcement leaders were genuinely surprised to learn that a syringe access law with such provisions for immunity from criminal paraphernalia possession was in effect at all (Melissia Larson, NCHRC Law Enforcement Liaison, personal communication, April 20, 2022). In addition to establishing clear department-level policies, efforts to educate officers on harm reduction efforts may be useful in limiting the harmful effects of punitive responses to syringe possession [39]. These efforts should be carefully crafted to include the lived experiences of law enforcement officers, lest they risk exacerbating negative law enforcement attitudes towards PWUD [39, 40].

Our findings also demonstrate how imperative it is that race be considered in research on the street-level decision-making of law enforcement. Our finding that law enforcement behavior disproportionately disadvantages Black PWUD evidences a through-line of anti-Black racism in drug policy and the leveraging of drug policy to criminalize Black persons [22, 28]. It also affirms previous scholarship that has seen law enforcement’s claim to their professional role as “fixers” of illicit drug problems continue unchallenged, even as public policy has shifted to favor compassionate, public health approaches to substance use [28].

Further, our finding that Black SSP participants are significantly more likely to report law enforcement doubting the ownership of their SSP cards parallels historical trends related to the contesting of Black property ownership. While NC’s syringe access law only requires that participants present written verification that the materials from an SSP were obtained from an SSP [6], participants regularly reported that law enforcement challenged the validity of SSP participant cards according to determinations of “ownership.” This may arguably be an over-interpretation of the statute and may constitute a violation of its provisions. Reliance on ideas of “ownership” to contest a legally protected activity invokes concepts from both property and criminal law. Legal scholars Taja-Nia Henderson and Jamila Jefferson-Jones have argued that attempts to use law enforcement to remove Black individuals from public and private property invoke the property “right to exclude” based on the implicit claim of “Blackness as nuisance” [41]. When Black individuals are considered a nuisance, individuals frame questions of ownership in a way that allows for exclusion. Here, law enforcement may—knowingly or unknowingly—use the question of ownership as a tool to exclude Black PWUD from legal protections afforded by the syringe access law.

The doubting of whether SSP participant cards in the possession of Black people “belong” to them is further complicated by the inequity Black communities face when accessing harm reduction services. Black communities face a myriad of barricades when attempting to access harm reduction services [42–44]. If law enforcement officers are aware of these access issues, they may question (consciously or subconsciously) whether Black participants have access to programs offering SSP services, which may motivate questioning of SSP identification card ownership. As to whether this outcome is truly a result of the knowledge of inequities faced by Black PWUD in the USA, misunderstanding of the law itself, anti-Black racism, or some other motivation, future work must be done to ascertain contextual factors that result in law enforcement officers finding utility in questioning whether the SSP card being presented “belongs” to the person holding it.

Overall, our findings provide further evidence that interactions with law enforcement remain an important social determinant of health for all PWUD in the USA, regardless of race or gender. There is a growing body of literature recognizing how inequity in the criminal-legal system produces inequity in community health [45–47]. While some studies have focused on more explicit acts of police violence, sociologist Rory Kramer called for research into the “slow violence” of policing which occurs when law enforcement actively hinders laws which are known to lead to better health outcomes [48]. As discussed above, police harassment leads to less utilization of health programs meant to assist PWUD [11–13] and worse health outcomes among PWUD (10, 34). In this way, the refusal to recognize SSP protections are a matter of life and death.

These findings are subject to certain limitations. All study participants were recruited through convenience sampling; thus, our study population may be subject to sampling bias. Broader application of these findings in regions of NC dissimilar to those included here or outside of NC may not be appropriate. The statistical analysis used here is insufficient to demonstrate causation without additional research. Our analyses may have been underpowered to detect significant differences in law enforcement interactions across race and/or gender. This is especially true for persons identifying with a racialized identity other than Black or White (including AI/AN, Asian, Hispanic, and mixed-race) who were under-represented in our sample and therefore could not be included in comparative analysis. Future research should prioritize the lived experiences and perspectives of these persons.

## Conclusion

Despite recent changes to state law granting SSP participants limited immunity from prosecution for the possession of SSP-obtained syringes, SSP participants in NC continue to experience negative and coercive law enforcement interactions under circumstances ostensibly falling within the purview of the state’s syringe access law. Of note, Black PWUD were significantly more likely to report a law enforcement officer doubting “ownership” of their SSP participant card despite the absence of any statutory language defining “ownership” or establishing it as a meaningful consideration in these cases. Enhanced education and training of law enforcement officers pertaining to changes in the SSP laws as well as closer attention from state leadership to officers’ implementation of these laws may be merited. Future research should further explore geographic variation in policing practices that may affect the efficacy of SSPs, with a focus on how those practices may differ across race and gender within those interactions.

## Data Availability

The datasets analyzed in this study are available from the corresponding author on reasonable request.

## Abbreviations

NC: North Carolina
SSP: Syringe service programs
PWUD: People who use drugs
NCHRC: North Carolina Harm Reduction Coalition
